# Ambient particulate matter pollution and stroke burden

**DOI:** 10.4103/mgr.MEDGASRES-D-25-00188

**Published:** 2026-09-12

**Authors:** Lu Jiang, Kuangyang Yu, Long Gu, Wenfeng Xiao, Wen Tan, Yong Jiang, Jianhua Peng

**Affiliations:** 1 Department of Neurosurgery, the Affiliated Hospital of Southwest Medical University, Luzhou, Sichuan Province, China; 2 Department of Neurosurgery, Chongqing University Fuling Hospital, Chongqing University, Chongqing, China; 3 Laboratory of Neurological Diseases and Brain Function, the Affiliated Hospital of Southwest Medical University, Luzhou, Sichuan Province, China; 4 Institute of Brain Science, Southwest Medical University, Luzhou, Sichuan Province, China; 5 Sichuan Clinical Research Center for Neurosurgery, the Affiliated Hospital of Southwest Medical University, Luzhou, Sichuan Province, China; 6 Academician (Expert) Workstation of Sichuan Province, the Affiliated Hospital of Southwest Medical University, Luzhou, Sichuan Province, China

**Keywords:** ambient particulate matter pollution, concentration index, disability-adjusted life years, GBD database, health inequalities, Global Burden of Disease, prediction, socio-demographic index, stroke, trends

## Abstract

As a major component of air pollution, ambient particulate matter pollution has rarely been studied with respect to its global stroke burden. This study aims to evaluate the global stroke incidence, mortality, and health inequalities related to ambient particulate matter pollution from 1990 to 2021. Utilizing the Global Burden of Disease Study 2021, we analyzed global stroke-related mortality, disability-adjusted life years, and their age-standardized rates. The estimated annual percentage change was calculated using a linear regression model. Health inequalities were measured using the concentration index and slope index of inequality. Between 1990 and 2021, global ambient particulate matter pollution-related stroke deaths and disability-adjusted life years increased by 107% and 101% respectively, yet age-standardized rates declined. China experienced the largest increases in deaths and disability-adjusted life years, Vietnam had the fastest rise in age-standardized mortality rates, the Russian Federation saw the steepest declines in deaths and disability-adjusted life years, and Estonia had the most rapid drop in age-standardized mortality rate. Middle socio-demographic index locations accounted for 46% of the global stroke burden, with age-standardized mortality rates and age-standardized disability-adjusted life years surpassing those in high socio-demographic index locations. From 1990 to 2021, age-standardized disability-adjusted life years rose in low-middle socio-demographic index locations but fell in high socio-demographic index locations. While the concentration index signaled an improvement in global inequality, the slope index of inequality in 2021 worsened compared with that in 1990. Between 1990 and 2021, the global burden of ambient particulate matter pollution-related stroke declined. However, substantial disparities persisted across different regions, sexes, and nations. For ongoing and future research efforts, priority should be given to high-risk populations and regions, while also developing innovative interventions to further reduce the incidence of ambient particulate matter pollution-related stroke and enhance patient outcomes.

## Graphical abstract

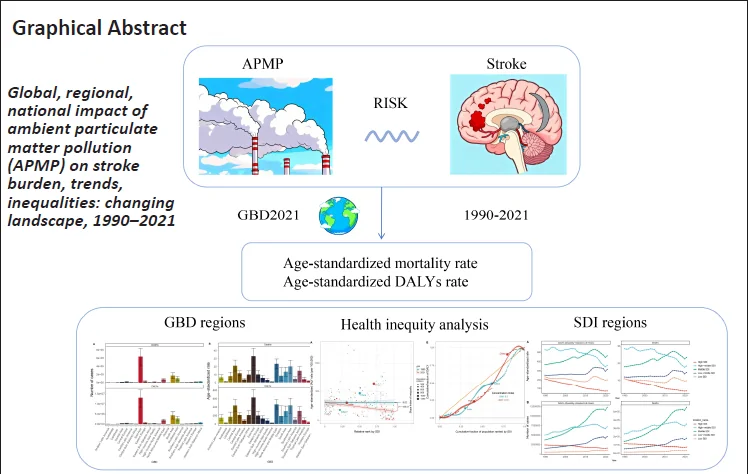

## Introduction

Previous studies have confirmed that among all risk factors for stroke, air pollution is a modifiable one, and reducing air pollution can decrease the incidence of stroke.^1^,^2^,^3^ Current hypotheses on the impact of air pollution on stroke include mechanisms such as direct ischemic damage to cerebral vessels or endothelial dysfunction caused by air pollutants, which can trigger cerebral vasospasm and autonomic dysfunction, and even intracranial pressure spikes from pollutant-induced coughing, potentially leading to stroke.^4^ Although these hypotheses are biologically plausible, most existing studies rely on associations between regional average pollution levels and disease occurrence, lacking comprehensive data to support definitive causal conclusions.

Given the high mortality and disability rates of stroke and its substantial societal burden, and since air pollution is a known risk factor for stroke, with ambient particulate matter pollution (APMP) as a core component and primary concern in air pollution, clarifying the relationship between APMP and stroke occurrence is critical for disease prevention and control.^4^,^5^ A previous study has shown that APMP ranks fourth among the factors influencing the global stroke burden, accounting for 20.2% of the stroke burden in 2019.^6^ The number of deaths attributed to APMP exceeds 4 million annually.^7^,^8^ Notably, if the air quality in all countries were to meet the guidelines set by the World Health Organization, human life expectancy is expected to increase by more than 14 years, which fully highlights the significant importance of reducing APMP concentrations.^9^ From 1990 to 2019, the stroke burden attributed to APMP showed an upward trend.^10^ Meanwhile, reduced precipitation caused by climate change may further exacerbate APMP.^11^ Therefore, sustained attention and in-depth research on APMP are essential. Utilizing the most recent Global Burden of Disease Study (GBD) 2021 data, this study analyzed the research gap regarding the impact of APMP on stroke burden from 1990 to 2021, assessed the global stroke burden attributable to APMP in air pollution. Further analysis is categorized by age, sex, countries, socio-demographic index (SDI) and GBD regions. Spatial and temporal trends, as well as health inequities across different SDI locations in APMP-related stroke burden, are investigated. This study aims to provide a statistical basis for health professionals and policymakers to implement large-scale actions to reduce stroke burden and socioeconomic costs, offering evidence for precise prevention and control strategies.

## Methods

### Data source

Data were retrieved from the GBD 2021 (https://ghdx.healthdata.org/gbd-2021), which provides annual estimates of disease burden for 371 diseases and related risk factors across 204 countries and territories since 1990. This study accessed data via the Global Health Data Exchange platform, including annual counts and age-standardized rates (ASRs; per 100,000 population) for stroke-related deaths years and disability-adjusted life years (DALYs). All data were stratified by sex, age group, geographical region, country, and SDI classification to allow for demographic and regional comparisons. As the study used publicly available aggregated data without individual identifiers, ethical approval and informed consent were not required.

### Definition

Stroke was operationally defined as the acute onset of focal neurological deficits persisting for more than 24 hours or leading to fatality, in the absence of an alternative etiology.^12^,^13^ In the context of air pollution, this study concentrated solely on APMP as the exposure of interest in estimating the attributable stroke burden.

The primary outcome measures were the age-standardized mortality rate (ASMR) and age-standardized DALY rate (ASDR), both reported per 100,000 population and adjusted for age distribution. Total DALYs combine years of life lost due to premature mortality with years lived with disability, reflecting the overall disease burden. The SDI, used to stratify countries and regions, is a composite indicator incorporating income per capita, average educational attainment, and fertility rate under 25-year-old. SDI values range from 0 to 1 and were divided into quintiles to classify countries into five levels of development: high, high-middle, middle, low-middle, and low SDI.

### Statistical analysis

Temporal trends in stroke burden attributable to APMP from 1990 to 2021 were assessed using a natural logarithm linear regression model, with the calendar year as the independent variable. The model takes the form:

ln(rate) = α + β × year

where α represents the intercept and β is the slope coefficient. The estimated annual percentage change (EAPC) was derived using the formula EAPC = (e^β^ − 1) × 100%, providing a summary measure of average annual change over time. A 95% confidence interval was calculated for each EAPC to assess the direction and significance of temporal trends.

To assess socioeconomic inequality in stroke burden attributable to APMP, we used the slope index of inequality (SII) and the concentration index (CI). The SII measures absolute differences in rates across the SDI spectrum, while the CI quantifies relative inequality based on the distribution of outcomes by socioeconomic rank. CI values range from –1 to +1, with negative values indicating a higher burden in lower-SDI populations and positive values indicating a concentration in higher-SDI groups. For all indicators, a value of zero reflects perfect equality.

All outcome estimates were accompanied by 95% uncertainty intervals (UI), derived from the 2.5% and 97.5% of 1000 posterior simulations, accounting for uncertainties related to data sources, model assumptions, and population heterogeneity. All data processing and analyses were conducted using R software (version 4.3.3; https://www.r-project.org).

## Results

### The ambient particulate matter pollution associated with global stroke burden in 2021

In 2021, 1,230,852 deaths (95% UI, 816,144–1,593,749) were attributed to APMP-related stroke, with the ASMR of 30.35 (95% UI, 20.24–39.25) per 100,000 person-years (**Table 1**). The DALYs due to APMP-related stroke in 2021 were 26,779,431 (95% UI, 17,663,527–34,439,115), with the ASDR of 636.05 (95% UI, 421.03–818.12) per 100,000 person-years (**Table 2**).

**Table 1:** The global number, ASDR, and temporal trends of deaths attributable to ambient particulate matter pollution-related stroke in 1990 and 2021

Trait		1990		2021		1990–2021
		
		Number of death cases (95% UI)	ASDR/100000 (95% UI)	Number of death cases (95% UI)	ASDR/100000 (95% UI)	EAPC (95% CI)
Global		594570 (410656 to 816224)	17.42 (12.14 to 23.95)	1230852 (834767 to 1575049)	14.78 (10.05 to 18.92)	–0.37 (–0.5 to –0.24)
Sex						
	Female	301533 (209694 to 417003)	15.52 (10.81 to 21.49)	546876 (355546 to 710729)	11.71 (7.61 to 15.22)	–0.83 (–0.93 to –0.73)
	Male	293037 (197900 to 414330)	19.68 (13.36 to 27.35)	683976 (460598 to 883020)	18.64 (12.63 to 24.03)	0.06 (–0.12 to 0.24)
Age (yr)						
	25–29	2284 (1511 to 3147)	0.52 (0.34 to 0.71)	2607 (1755 to 3357)	0.44 (0.3 to 0.57)	–0.19 (–0.38 to 0.01)
	30–34	3519 (2348 to 4905)	0.91 (0.61 to 1.27)	5195 (3509 to 6657)	0.86 (0.58 to 1.1)	–0.02 (–0.16 to 0.12)
	35–39	5970 (3934 to 8452)	1.69 (1.12 to 2.4)	8670 (5908 to 11128)	1.55 (1.05 to 1.98)	–0.18 (–0.35 to –0.01)
	40–44	9438 (6328 to 13370)	3.29 (2.21 to 4.67)	15146 (10218 to 19510)	3.03 (2.04 to 3.9)	–0.09 (–0.3 to 0.12)
	45–49	13712 (9168 to 19178)	5.91 (3.95 to 8.26)	25434 (16994 to 32794)	5.37 (3.59 to 6.93)	0.02 (–0.23 to 0.27)
	50–54	24837 (16631 to 35481)	11.68 (7.82 to 16.69)	45526 (29889 to 58675)	10.23 (6.72 to 13.19)	–0.25 (–0.42 to –0.08)
	55–59	36069 (23904 to 51527)	19.48 (12.91 to 27.82)	68334 (44666 to 88442)	17.27 (11.29 to 22.35)	–0.33 (–0.46 to –0.2)
	60–64	54192 (36445 to 75556)	33.74 (22.69 to 47.04)	91533 (61006 to 117287)	28.6 (19.06 to 36.65)	–0.31 (–0.49 to –0.13)
	65–69	68285 (46074 to 96185)	55.24 (37.27 to 77.81)	141464 (94020 to 182595)	51.28 (34.08 to 66.2)	–0.31 (–0.45 to –0.17)
	70–74	80272 (54227 to 113739)	94.82 (64.05 to 134.35)	179209 (118792 to 229244)	87.06 (57.71 to 111.37)	–0.24 (–0.41 to –0.06)
	75–79	103758 (72680 to 142800)	168.56 (118.07 to 231.99)	188872 (126475 to 240748)	143.21 (95.9 to 182.54)	–0.21 (–0.39 to –0.03)
	80–84	95875 (68082 to 131257)	271.02 (192.45 to 371.04)	196360 (135166 to 251038)	224.2 (154.33 to 286.63)	–0.31 (–0.48 to –0.15)
	85–89	64631 (46060 to 89027)	427.71 (304.81 to 589.15)	163316 (113022 to 208162)	357.19 (247.19 to 455.28)	–0.44 (–0.57 to –0.31)
	90–94	24741 (17242 to 34660)	577.35 (402.35 to 808.83)	76025 (52881 to 98031)	424.97 (295.6 to 547.99)	–0.91 (–1.03 to –0.79)
	95+	6986 (4671 to 9908)	686.15 (458.79 to 973.24)	23163 (15583 to 30685)	424.98 (285.91 to 562.99)	–1.65 (–1.88 to –1.43)

ASDR: Age-standardized disability-adjusted life years; CI: confidence interval; EAPC: estimated annual percent change; UI: uncertainty intervals.

**Table 2:** The global number, ASDR, and temporal trends of DALYs attributable to air pollution-related stroke in 1990 and 2021

Trait		1990		2021		1990–2021
		
		Number of death cases (95% UI)	ASDR/100,000 (95% UI)	Number of death cases (95% UI)	ASDR/100,000 (95% UI)	EAPC (95% CI)
Global		13337927 (9219590 to 18469720)	349.71 (241.1 to 483.16)	26779431 (18076188 to 34223093)	312.85 (211.45 to 399.79)	–0.2 (–0.34 to –0.07)
Sex						
	Female	6164218 (4226821 to 8571372)	297.54 (204.33 to 412.85)	11247883 (7258687 to 14524263)	244.08 (157.48 to 315.14)	–0.56 (–0.66 to –0.45)
	Male	7173709 (4792684 to 10211357)	408.2 (275.13 to 578.09)	15531548 (10404840 to 19914852)	391.97 (263.55 to 502.98)	0.08 (–0.09 to 0.26)
Age (yr)						
	25–29	173466 (116835 to 243022)	39.19 (26.4 to 54.91)	209742 (140324 to 272488)	35.65 (23.85 to 46.31)	0.01 (–0.18 to 0.21)
	30–34	238159 (160785 to 332965)	61.79 (41.72 to 86.39)	367919 (252059 to 476702)	60.87 (41.7 to 78.86)	0.13 (0 to 0.26)
	35–39	357638 (238286 to 503895)	101.53 (67.65 to 143.05)	541899 (368644 to 701031)	96.62 (65.73 to 124.99)	–0.04 (–0.2 to 0.12)
	40–44	498633 (337323 to 706009)	174.05 (117.75 to 246.44)	825674 (562894 to 1054095)	165.05 (112.52 to 210.71)	0.02 (–0.18 to 0.22)
	45–49	643197 (435438 to 891844)	277.01 (187.53 to 384.09)	1234734 (831381 to 1581269)	260.76 (175.58 to 333.95)	0.13 (–0.11 to 0.37)
	50–54	1026032 (693821 to 1470651)	482.68 (326.39 to 691.84)	1939364 (1285422 to 2494136)	435.89 (288.91 to 560.58)	–0.13 (–0.3 to 0.04)
	55–59	1300936 (870282 to 1871400)	702.45 (469.91 to 1010.48)	2545521 (1678709 to 3281459)	643.25 (424.21 to 829.22)	–0.22 (–0.35 to –0.09)
	60–64	1675014 (1135506 to 2349690)	1042.91 (707 to 1462.99)	2903468 (1954495 to 3712981)	907.2 (610.69 to 1160.13)	–0.21 (–0.39 to –0.02)
	65–69	1770246 (1205718 to 2486826)	1432.13 (975.43 to 2011.84)	3765655 (2511165 to 4824692)	1365.15 (910.36 to 1749.08)	–0.21 (–0.35 to –0.07)
	70–74	1700377 (1151945 to 2395461)	2008.45 (1360.65 to 2829.47)	3904097 (2605784 to 4992082)	1896.67 (1265.93 to 2425.23)	–0.15 (–0.3 to 0.01)
	75–79	1745420 (1224721 to 2394235)	2835.52 (1989.62 to 3889.55)	3281733 (2207107 to 4191938)	2488.34 (1673.52 to 3178.49)	–0.11 (–0.29 to 0.06)
	80–84	1259370 (895958 to 1715107)	3559.96 (2532.68 to 4848.23)	2647981 (1830046 to 3358808)	3023.39 (2089.5 to 3835)	–0.22 (–0.39 to –0.05)
	85–89	669204 (474480 to 921796)	4428.57 (3139.95 to 6100.13)	1720847 (1189327 to 2188942)	3763.74 (2601.23 to 4787.52)	–0.38 (–0.51 to –0.26)
	90–94	221281 (154240 to 309614)	5163.87 (3599.37 to 7225.21)	692216 (485389 to 890831)	3869.43 (2713.28 to 4979.67)	–0.86 (–0.98 to –0.73)
	95+	58954 (39626 to 83572)	5790.64 (3892.18 to 8208.73)	198582 (134063 to 262122)	3643.49 (2459.73 to 4809.3)	–1.62 (–1.84 to –1.39)

ASDR: Age-standardized disability-adjusted life years; CI: confidence interval; DALYs; disability-adjusted life years; EAPC: estimated annual percent change; UI: uncertainty intervals.

### The ambient particulate matter pollution associated with global stroke burden by age in 2021.

DALYs were highest in the 70–74 age group, followed by the 65–69 age group (**Figure 1**). Stratified by age, the ASMR of APMP-related stroke in 2021 increased with age. The ASDR peaked in the 90–94 age group. In terms of case number, the highest mortality occurred in the 80–84 age group, followed by the 75–79 and 70–74 age groups.

**Figure 1:**
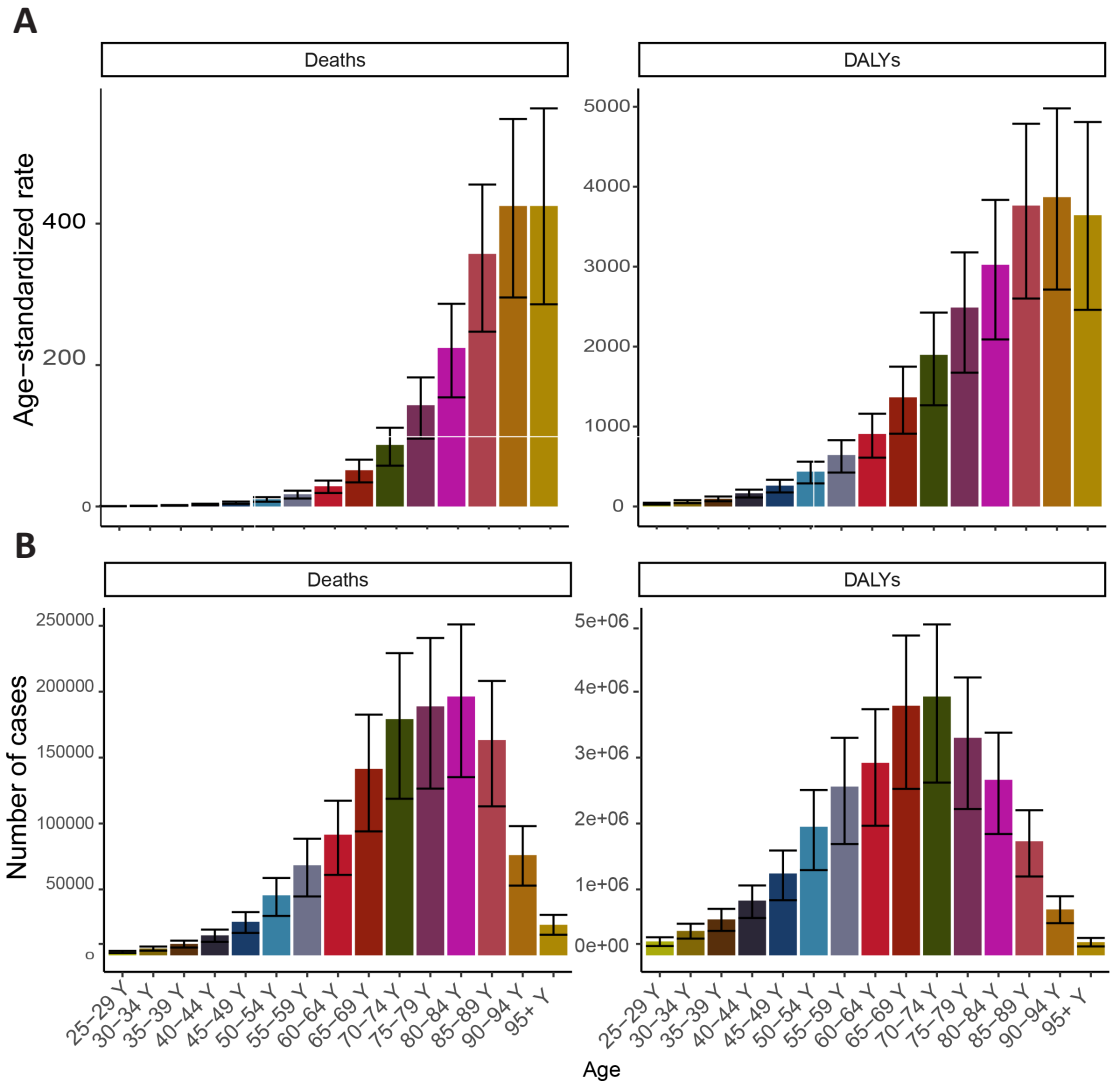
Cases and age-standardized rates of deaths and DALYs attributable to ambient particulate matter pollution-related stroke grouped by age in 2021. (A) Age-standardized rates of deaths and DALYs in 2021. (B) Cases of deaths and DALYs in 2021. DALYs: Disability-adjusted life years.

### The ambient particulate matter pollution associated with global stroke burden by sex in 2021

Regarding the sex differences in stroke burden in 2021, the ASMR of APMP-related stroke was 18.64 (95% UI, 12.63–24.03) for males and 11.71 (95% UI, 7.61–15.22) for females. Male deaths were nearly 25% higher than female deaths (males: 683,976, 95% UI, 460,598–883,020; females: 546,876, 95% UI, 355,546–710,729). The DALYs were 15,531,548 (ASDR, 391.97, 95% UI, 263.55–502.98) for males and 11,247,883 (ASDR, 244.08, 95% UI, 157.48–315.14) for females.

### The ambient particulate matter pollution associated with global stroke burden by Global Burden of Disease Study regions in 2021

Among the 21 GBD regions, East Asia had the highest number of APMP-related stroke deaths (645,929, 95% UI, 413,797–844,249) and the highest DALYs (13,188,663, 95% UI, 8,474,561–17,093,130). When considering ASMR and ASDR, East Asia (ASMR: 33, 95% UI, 21.27–42.99) (ASDR: 627.8, 95% UI, 404.82–813.83) remained the highest-ranked (**Figure 2**). Other notable regions included North Africa and the Middle East (ASMR: 23.48, 95% UI, 17.73–29.41; ASDR: 463.54, 95% UI, 350.54–575.62), Central Asia (ASMR: 21.01, 95% UI, 14.56–28.05), and Southeast Asia (ASDR: 440.87, 95% UI, 257.14–607.71), each region had relatively high ASMR and ASDR.

**Figure 2:**
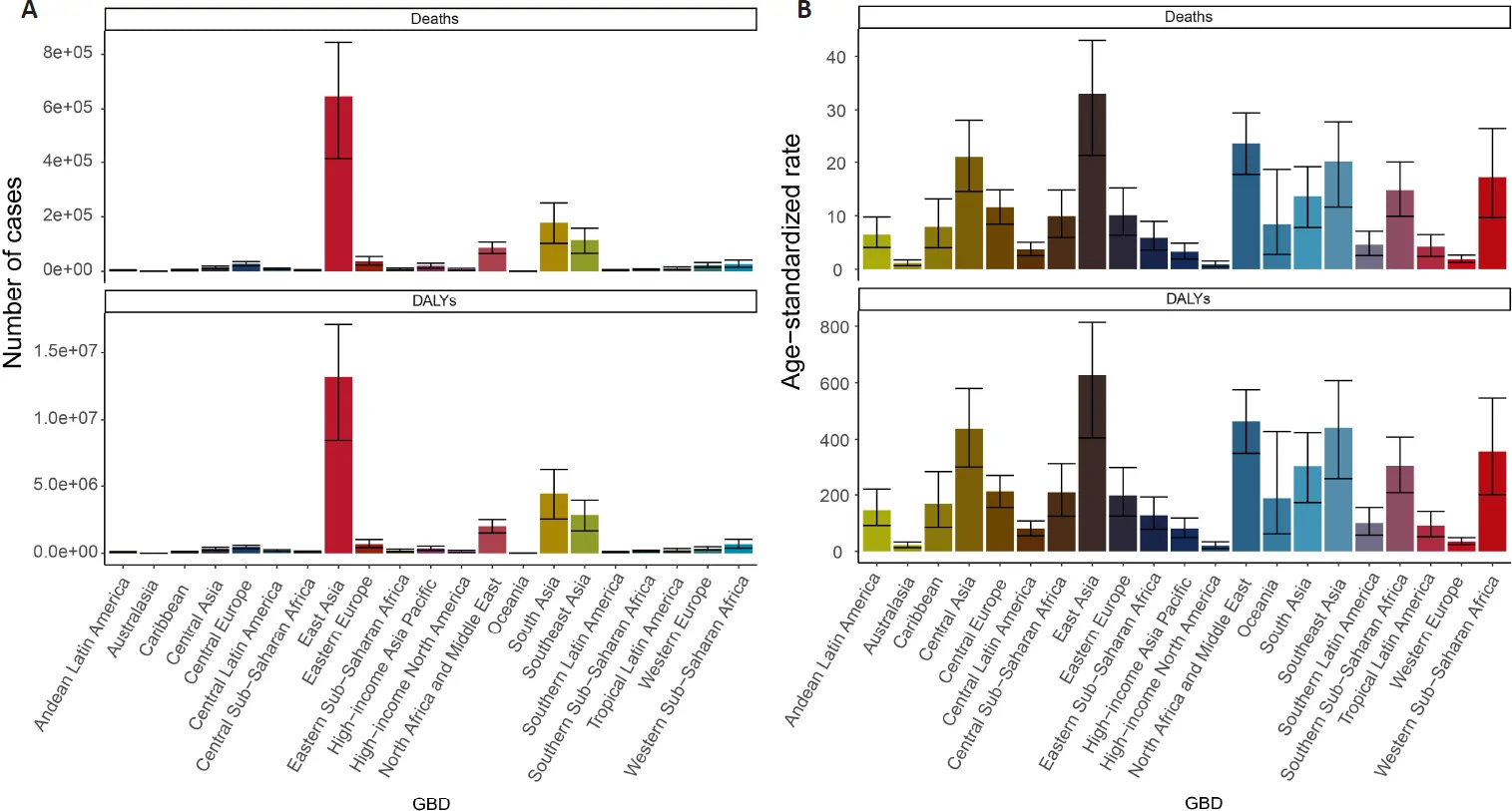
Cases and age-standardized rates of deaths and DALYs attributable to ambient particulate matter pollution-related stroke across GBD regions. (A) Cases of deaths and DALYs across GBD regions. (B) Age-standardized rates of deaths and DALYs across GBD regions. DALYs: Disability-adjusted life years; GBD: Global Burden of Disease Study.

### The ambient particulate matter pollution associated with global stroke burden by countries and territories in 2021

In 2021, the burden of APMP-related stroke was also analyzed across 204 countries and territories. Among these 204 countries and territories, Egypt had the highest age-standardized death rate (ASMR: 66.26, 95% UI, 46.68–86.59), followed by North Macedonia (AMSR: 51.69, 95% UI, 33.52–69.05) and Iraq (ASMR: 44.71, 95% UI, 30.61–61.5). Egypt also had the highest ASDR (ASDR: 1222.61, 95% UI, 864.22–1588.03), followed by Iraq (ASDR: 856.53, 95% UI, 574.44–1187.85) and North Macedonia (ASDR: 790.61, 95% UI, 509.97–1058.8). Iceland had the lowest AMSR (0.33, 95% UI, 0.06–0.75) and ASDR (6.45, 95% UI, 1.19–14.28).

### The ambient particulate matter pollution associated with global stroke burden by socio-demographic index in 2021

Stroke burden characteristics were analyzed globally by SDI stratification. Cross-sectional data in 2021 showed that high SDI locations had an ASR of 3.44 (95% confidence interval, 2.56–4.49) and ASDR of 79.95 (95% confidence interval, 61.36–101.85), while middle-SDI locations had much higher ASR and ASDR at 23.5 (95% confidence interval, 14.7–30.47) and 472.1 (95% confidence interval, 297.91–602.67), respectively. Middle-SDI locations also dominated in case numbers (561,417, 95% confidence interval, 352,627–725,345) and DALYs (12,300,098, 95% confidence interval, 7,781,567–15,739,017), accounting for 46% of the global total.

### The trends of ambient particulate matter pollution-related stroke burden from 1990 to 2021

Globally, the number of APMP-related stroke deaths increased by 107%, from 594,570 (95% UI, 407,594–831,333) in 1990 to 1,230,852 in 2021, with some fluctuations. However, the AMSR decreased from 35.2 (95% UI, 24.17–48.84) in 1990 to 30.35 (95% UI, 20.24–39.25) in 2021. The number of DALYs rose by approximately 101%, from 13,337,927 (95% UI, 9,019,505–18,782,729) in 1990 to 26,779,431 in 2021. Despite this, the ASDR declined from 705.74 (95% UI, 479.46–990.94) in 1990 to 636.05 (95% UI, 421.03–818.12) in 2021. Notably, a slight increase in APMP-related stroke burden was observed between 2020 and 2021 (**Figure 3**).

**Figure 3:**
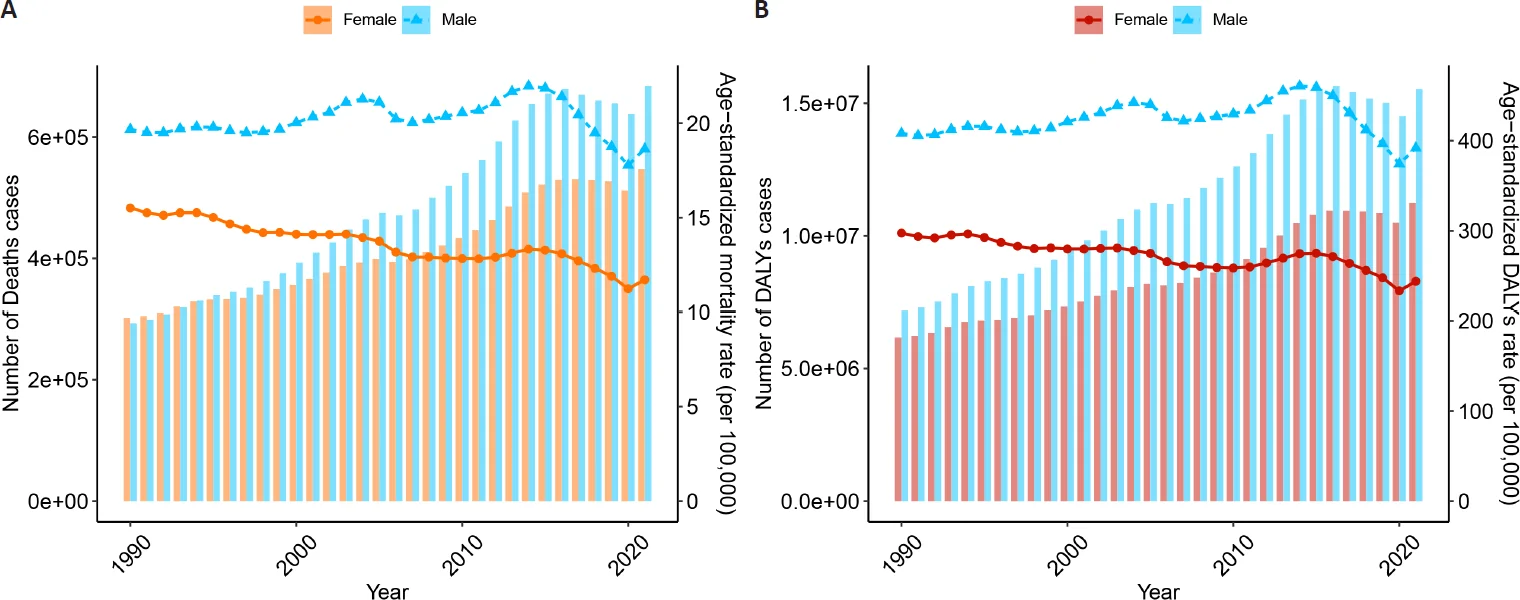
Temporal trends of the number of deaths, age-standardized mortality rates, the number of DALYs and age-standardized DALYs rates of ambient particulate matter pollution-related stroke by sex from 1990 to 2021. (A) Temporal trends of the number of deaths and age-standardized mortality rates of ambient particulate matter pollution-related stroke by sex from 1990 to 2021. (B) Temporal trends of the number of DALYs and age-standardized DALYs rates of ambient particulate matter pollution-related stroke by sex from 1990 to 2021. DALYs: Disability-adjusted life years.

### The trends of ambient particulate matter pollution-related stroke burden by age from 1990 to 2021

Consistent with global trends, the AMSR for age groups over 80 and the ASDR for age groups over 70 declined. Compared with 1990, deaths and DALYs increased in 2021, with rises in deaths and DALYs in the age groups over 40. The number of deaths increased most in the age groups over 95 (231%), while DALYs rose most in the 85–89 age group (157%). The 70–74 age group showed steady DALY growth (130%). All age groups demonstrated declining trends in ASR and ASDR (**Figure 4**). Sex subgroup trends reflected the global pattern, with males having higher deaths and DALYs than females (**Figure 3**).

**Figure 4:**
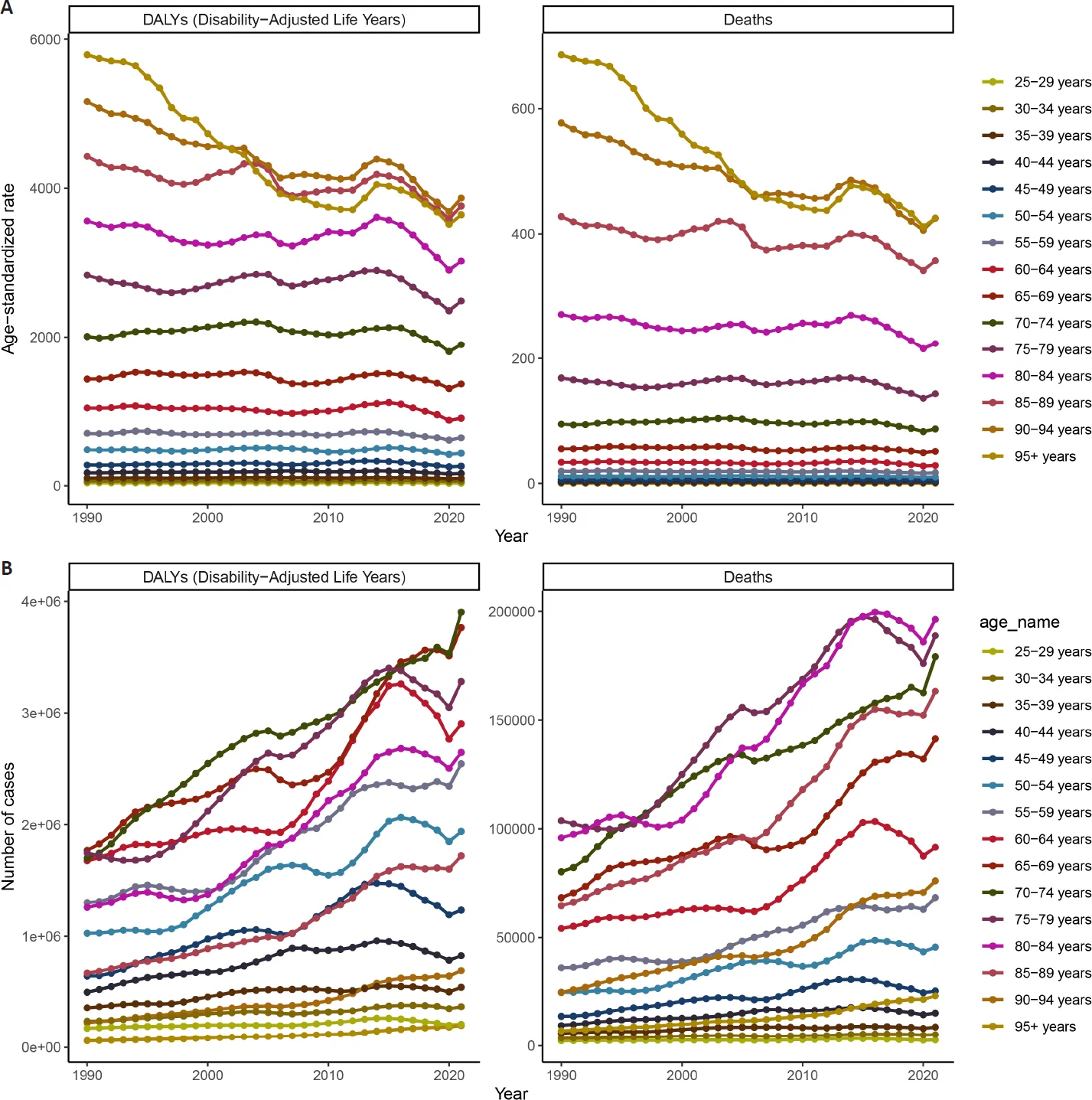
Temporal trends of age-standardized rates and number of cases for deaths and DALYs attributable to ambient particulate matter pollution-related stroke across age groups from 1990 to 2021. (A) Temporal trends of age-standardized rates of deaths and DALYs from 1990 to 2021. (B) Temporal trends of the number of cases of deaths and DALYs from 1990 to 2021. DALYs: Disability-adjusted life years.

### The trends of ambient particulate matter pollution-related stroke burden by countries and territories from 1990 to 2021

Further analysis at the country and territory level revealed that China had the most pronounced increase in the number of death cases (327.69%), while the Russian Federation showed the largest decrease (–174.69%). China also had the largest increase in DALYs (9,465,070), whereas the Russian Federation had the largest increase in DALYs (853,302). The most dramatic yearly increment in AMSR was in Vietnam, with an EAPC of 4.95 (95% confidence interval, 4.6–5.29), while Estonia showed the greatest annual decrease, with an EAPC of –10.72 (95% confidence interval, –11.43 to –10). For ASDR, Vietnam again had the highest annual growth with an EAPC of 4.81 (95% confidence interval, 4.46–5.17), in contrast to Estonia showed the largest annual decline with an EAPC of -10.33 (95% confidence interval, –10.98 to –9.66).

### The trends of ambient particulate matter pollution-related stroke burden by socio-demographic index from 1990 to 2021

From 1990 to 2021, the socioeconomic inequality in stroke burden attributable to APMP showed a divergent trend. The SII declined from 9.02 to –155.51, indicating a substantial increase in absolute inequality, with a growing burden among lower-SDI populations. In contrast, the CI decreased slightly from 0.20 to 0.16, suggesting a modest reduction in relative inequality, although the burden remained disproportionately concentrated in higher-SDI regions. This divergence shows the need to interpret absolute and relative inequality measures in parallel when evaluating environmental health disparities (**Figure 5**).

**Figure 5:**
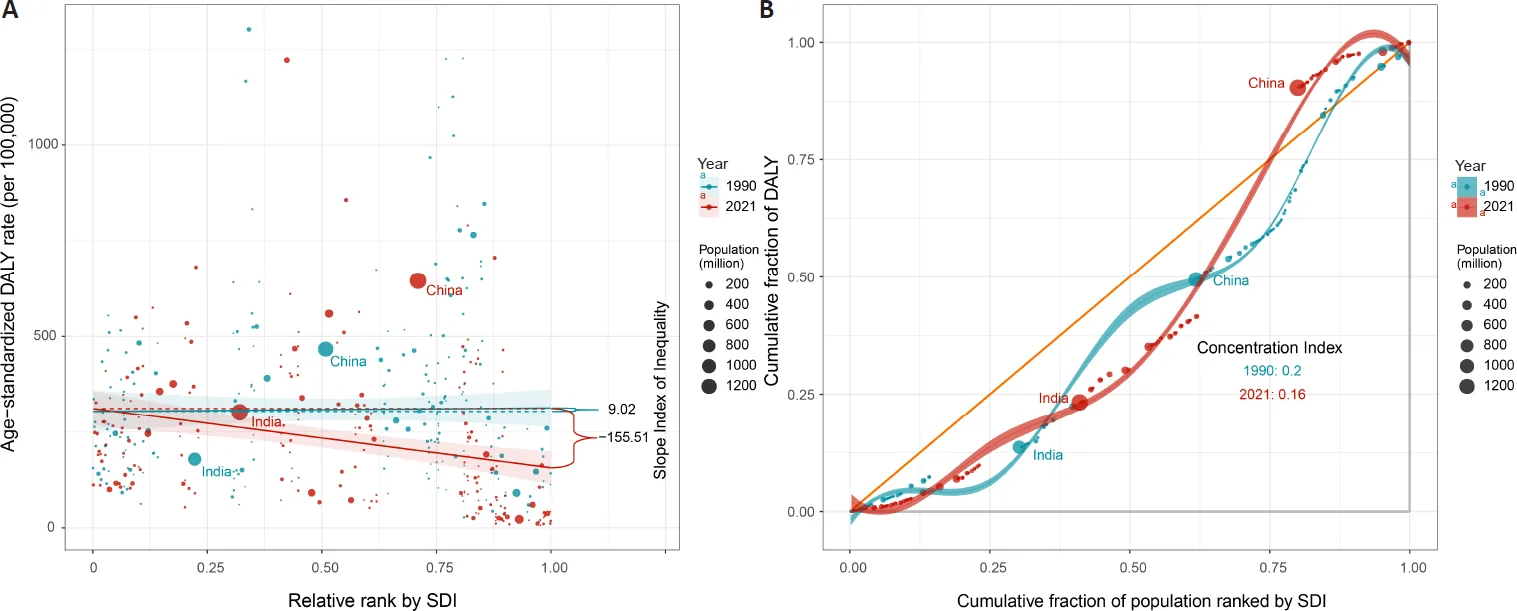
The 1990–2021 absolute and relative transnational inequalities of ASDR related to ambient particulate matter pollution-associated stroke. (A) Health inequality regression curves of ASDR related to ambient particulate matter pollution-associated stroke. (B) Concentration curves of ASDR related to ambient particulate matter pollution-associated stroke. ASDR: Age-standardized disability-adjusted life years.

Trends from 1990 to 2021 indicated that middle- and low-middle-SDI locations experienced continuous increases in ASR and ASDR, while all other regions saw declines. High-SDI locations exhibited reductions in the number of deaths and DALYs, whereas all other regions showed increases (**Figure 6**).

**Figure 6:**
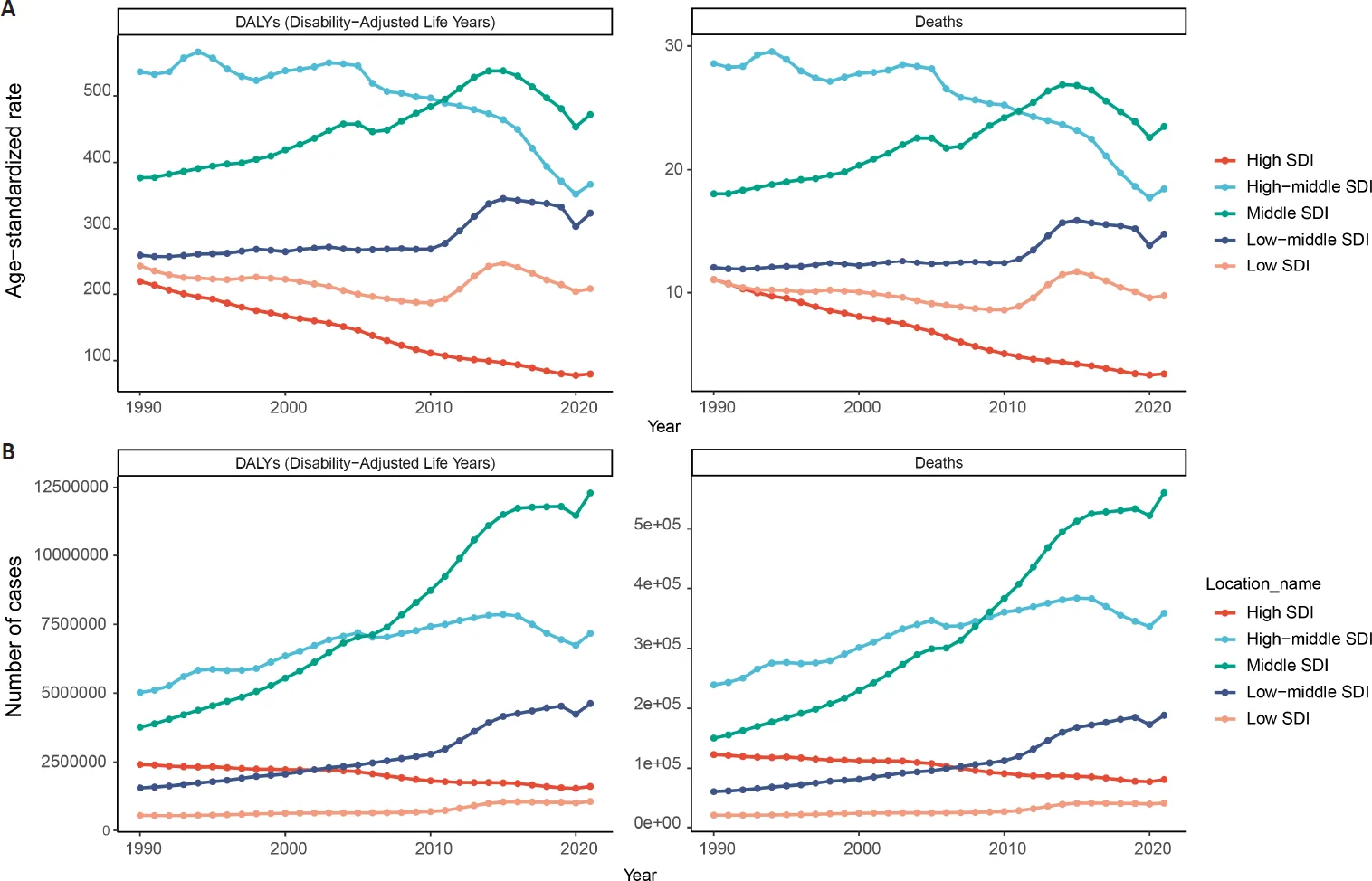
Trends of ASDR, ASMR, DALYs, and number of deaths related to ambient particulate matter pollution-associated stroke across different SDI regions during 1990–2021. (A) Trends of ASDR and ASMR for ambient particulate matter pollution-associated stroke. (B) Trends of DALYs and number of deaths for ambient particulate matter pollution-associated stroke. ASDR: Age-standardized disability-adjusted life years; ASMR: age-standardized mortality rates; DALYs; disability-adjusted life years; SDI: Socio-demographic Index.

## Discussion

APMP, a key component of air pollution, is one of the modifiable risk factors for stroke worldwide.^14^ Given its high mortality and disability rates, stroke presents a significant public health challenge. However, previous studies have predominantly been confined to specific regions, short time frames, or single-dimensional analyses, with insufficient investigation into the long-term spatiotemporal evolution of the global APMP-related stroke burden since 1990, health inequalities across regions and countries with varying SDI, and changes in ASRs and absolute burden. Moreover, there has been a lack of systematic assessment based on the GBD 2021 data.^15^ Addressing this research gap, the present study systematically analyzed the global burden, spatiotemporal evolution characteristics, and health inequalities of APMP-related stroke from 1990 to 2021, utilizing GBD 2021 data. The results indicated that despite a downward trend in both AMSR and ASDR from 1990 to 2021, the number of deaths and DALYs continued to rise. The decline in AMSR and ASDR may be attributed to advancements in modern medical technology, environmental enhancements, early stroke prevention strategies, and progress in related management practices. Nevertheless, due to the worsening of population aging, the number of stroke-related deaths and DALYs still increased, implying that while pollution control measures have yielded some success, the expanding elderly population has partly negated these advancements. Future initiatives should focus on bolstering environmental governance and health management for high-risk groups.^16^,^17^,^18^,^19^

Recent studies indicate that air pollutants may increase the risk of stroke through multiple mechanisms. Short-term exposure can induce systemic inflammatory responses and oxidative stress, damaging the vascular endothelium, while long-term exposure is associated with elevated blood pressure and an increased risk of vascular rupture due to arterial vasoconstriction.^4^,^20^,^21^ APMP can enter the systemic circulation via direct translocation, cross the blood-brain barrier to reach the brain, and induce pathophysiological changes in the central nervous system through its own physical properties or by carrying toxic compounds, thereby increasing the risk of stroke. It may also trigger systemic inflammation and oxidative stress by initially recruiting immune cells in lung tissues, and neuroinflammation and cerebral oxidative stress are key factors in stroke.^22^,^23^,^24^,^25^,^26^,^27^,^28^,^29^ Furthermore, APMP can induce hemodynamic changes, such as increased blood pressure, arrhythmia, and vasoconstriction, leading to an imbalance in the autonomic nervous system and an elevated risk of stroke.^30^,^31^,^32^,^33^ Through these potential mechanisms, APMP collectively promotes the occurrence and progression of stroke, explaining why the stroke burden is higher in highly polluted regions such as East Asia and North Africa, as well as in populations with longer exposure durations like the elderly.

Notably, elevated concentrations of particulate matter at night cause more severe vascular endothelial dysfunction than exposure during the day, suggesting that circadian regulation plays a key role in the body’s response to pollution.^34^ Consistent with previous studies, this study found that age-standardized stroke mortality increases with age, peaking particularly in those over 95, with particular susceptibility among the elderly, possibly due to reduced vascular elasticity and higher prevalence of cardiovascular diseases in older adults.^17^,^35^ In terms of sex, this study indicates higher stroke mortality in males than females, which may be associated with increased occupational exposure and smoking-related confounders in men.^36^,^37^

In terms of region, East Asia experiences the highest stroke burden, likely linked to its rapid industrial development and severe particulate pollution.^38^,^39^ Regions such as East Asia, Southeast Asia, and North Africa exhibit a higher stroke burden than the global average, often encountering severe air pollution, rapid urbanization, and insufficient healthcare resources.^8^ At the national level, Egypt, Iraq, and North Macedonia exhibit higher AMSR and ASDR for stroke than other regions. This is closely linked to high levels of air pollutants and lower healthcare standards. Additionally these areas commonly rely on fossil-fuel-based energy structures and have high rates of indoor solid fuel use, which is consistent with previous research findings on household air pollution in China and India.^40^ In contrast, high-SDI locations such as Europe and North America, which have implemented stricter air quality control policies and advanced medical technologies, exhibit a lower stroke burden than the global average.^41^ Moreover, Nordic countries such as Iceland, which have widely adopted clean energy, exhibit the lowest stroke burden, confirming the positive impact of the energy transition on improving air quality and preventing strokes.^34^

To reduce APMP, regions with high burdens, such as East Asia and North Africa, could strive to decrease the consumption of fossil fuels, encourage the adoption of clean energy, foster a green industrial transformation, enhance public transportation systems, cut down on exhaust pollution, control dust pollution, bolster urban ecological conservation, improve monitoring and enforcement of environmental laws, ensure the execution of environmental protection policies, and conduct environmental protection publicity and education. In terms of treatment, these high-burden regions could emulate countries that have made substantial progress in stroke prevention and management. They could establish a telemedicine stroke emergency network and a collaborative hierarchical emergency system, and implement the execution and innovation of rehabilitation systems. For instance, China has drawn upon the stroke emergency system of Bavaria, Germany, and has jointly established a telemedicine ultra-early emergency network. Furthermore, each country should develop stroke intervention strategies that focus on managing high-risk factors and are tailored to its level of development, based on its unique circumstances. Targeted measures should be implemented for young people, the elderly, different sex, and regional characteristics.

Between 1990 and 2021, the global decline in age-standardized stroke rates was closely associated with air quality management policies. Research on Tokyo’s diesel emission control act demonstrated an obvious correlation between emission controls, air quality improvement, and reduced stroke incidence. After implementing China’s Air Pollution Prevention and Control Action Plan, key Chinese cities experienced notable declines in APMP alongside concurrent reductions in stroke-related mortality. These studies collectively indicate that pollution control policies can directly translate to significant reductions in stroke risk, providing empirical evidence for environmental governance and public health development.^42^,^43^

The high increase in stroke mortality in China may be related to accelerated aging, as stroke risk rises with age, coupled with the growing prevalence of chronic conditions like hypertension and diabetes, along with the cumulative effects of pollutant exposure during rapid urbanization. The largest rise in DALYs likely reflects that, despite improved acute stroke care reducing short-term mortality, the high disability rate of stroke leads to long-term health loss accumulation, compounded by insufficient rehabilitation coverage for survivors.^36^ The negative growth in Russia may be attributed to post-soviet heavy industry decline and stricter environmental regulations.^44^ Notably, Vietnam’s accelerated industrialization contributed to the fastest annual AMSR growth, highlighting the conflict between economic development and environmental health in developing countries.^10^ The SDI analysis reveals that stroke burden continues to rise in middle and low-middle SDI locations, while high-SDI locations achieve reduced disease burden. This contradiction highlights the close link between pollution control and energy structure, as high-SDI locations reduce pollution through clean energy transition, whereas middle and low-middle SDI locations face increased pollution exposure due to accelerated industrialization.^1^

Notably, the healthcare systems in low SDI countries are underdeveloped, and data from low SDI regions may be incomplete.^10^ Rural and remote areas lack diagnostic equipment such as CT and MRI, resulting in a high underreporting rate of stroke.^45^ The coverage of death registration systems is insufficient, and deaths related to strokes are often classified as having unspecified causes, leading to an underestimation of the actual burden. The number of neurologists is small, and the diagnostic capabilities of primary care physicians are limited, which may easily result in misdiagnosis and underreporting. The environmental monitoring system is incomplete, and the concentrations of APMP rely on satellite retrieval or model inference, which may affect the accuracy of the analysis. In contrast, countries with high SDI generally have established national disease surveillance networks, achieved data linkage through medical insurance systems, and promoted the improvement of pollution monitoring and disease registration systems in tandem with public health policies, resulting in higher timeliness and accuracy of data.

The current study has several limitations. It relies heavily on the GBD database, which complicates the accurate reporting of stroke-related deaths and other causes. This reliance may result in an underestimation of stroke mortality statistics and is constrained by issues with diagnosis and misclassification, further contributing to the underreporting of related data. Additionally, due to the absence of original data with detailed information, statistics on specific stroke causes are insufficient, hindering the investigation of their heterogeneous associations with environmental particulate pollutants. This study only analyzed overall stroke without conducting further subgroup analysis, which may lead to an underestimation of particulate matter’s contribution to specific pathological mechanisms. Because high-quality, high-resolution exposure estimates were unavailable, the influence of other air pollutants, meteorological factors, and environmental noise was not considered. Underreporting or misclassification in low SDI countries may narrow the burden gap with high SDI countries, and the actual health inequalities may be more significant. The stroke burden in low SDI countries needs to consider the potential underestimation caused by data underreporting.

Overall, from 1990 to 2021, the global burden of APMP-related stroke decreased, but substantial disparities existed across regions, sexes and countries. This study revealed an increasing trend of APMP-related stroke burden with age. It also showed higher APMP-related stroke burden among males than among females. Greater attention should focus on regions represented by East Asia, North Africa, the Middle East and Central Asia, and countries such as Egypt, North Macedonia and Iraq regarding the balance between air quality management and sustainable economic development. Ongoing and future research should prioritize high-risk populations and regions while developing innovative interventions to further reduce stroke incidence and improve outcomes.

## Data Availability

*Data were publicly available at https://vizhub.healthdata.org/gbd-results/.*
